# Treatment-seeking behaviour for childhood fever among caretakers of Chivuna and Magoye rural communities of Mazabuka District, Zambia: a longitudinal study

**DOI:** 10.1186/s12889-016-3460-8

**Published:** 2016-08-11

**Authors:** Benson M. Hamooya, Gershom Chongwe, Rosalia Dambe, Hikabasa Halwiindi

**Affiliations:** 1 Ministry of Health, P.O Box 30205, Lusaka, Zambia; 2 School of Medicine, Department of Public Health, The University of Zambia, P.O. Box 50110, Lusaka, Zambia

**Keywords:** Treatment-seeking, Childhood fever, Caretakers, Under-5 children

## Abstract

**Background:**

Treatment-seeking for childhood fever among caretakers in most rural parts of African region is still a major challenge. The aim of this study was to determine the treatment seeking behaviour for fever in under-5 children of Magoye and Chivuna rural areas of Mazabuka district in Zambia.

**Methods:**

Treatment-seeking behaviour was explored longitudinally among caretakers of 362 children aged 12–59 months with fever. The data was collected from caretakers using a structured interviewer-administered questionnaire at their homes. Chi-square test, one-sample test of proportions and logistic regression were the statistical methods used for data analysis.

**Results:**

Of the 362 children with fever, 77 % of them had their treatment sought externally. In which 64 % had their treatment at health facility (HF), 18 % from community health workers (CHW), and 18 % from other sources. Early treatment (≤ 24 h) was sought for 42 % of the fever episodes. In dry season, a child had 1.53 times more likely to have early treatment compared to rainy season [OR 1.53; 95 % CI 1.30, 1.80; *p* < 0.001]. A child in Chivuna was less likely to have early treatment compared to one in Magoye [OR 0.62; 95 % CI 0.50, 0.76; *p* < 0.001]. Caretakers had a reduced chance of 27 % [OR 0.73; 95 % CI 0.56, 0.95; *p* = 0.022] of seeking early treatment if they took a child to other sources compared to a HF.

**Conclusion:**

This study has revealed that seeking early and appropriate treatment was suboptimal in the study areas. Source of treatment, season and location were predictors of early treatment of fever among caretakers. Policies aimed at combating poor care-seeking behaviour should not omit to address these factors.

## Background

In recent past, most malaria endemic areas have used fever as a proxy for malaria even though the cause could be different [1]. Childhood fever is the common clinical sign of *P. falciparum* infection [2]. However, Malaria, pneumonia and diarrhoea are responsible for most of the morbidity and mortality in under-5 year old children worldwide, and accounts for approximately 3.6 million deaths annually [3]. Of these three causes, malaria and pneumonia have remained as the leading causes of morbidity and mortality in children under the age of five in sub-Saharan Africa [4]. A large proportion of malaria/fever cases are treated outside the formal health care system [5]. Because many rural areas have limited resources, health facilities are not easily accessible to much of the population [6], and as such most sick children end up being treated through the informal sector, including community health workers, drug sellers, and traditional healers [7]. This inadequacy of health care facilities, coupled with lack of skilled health care workers greatly impacts the treatment of many childhood illnesses and contributes to childhood morbidity and mortality.

It has been shown that timeliness in seeking fever treatment is significantly enhanced by caretakers’ knowledge of fever as a malaria symptom [8]. Furthermore, it has also been established that health care seeking behaviour in under-5 children is influenced by educational attainment, knowledge of the causes of malaria, age of the caretakers, availability of health facilities and daily activities of the caretakers [9–11].

In sub-Saharan Africa [12], it was revealed that community-based agents are the first people to attend to and treat childhood fever. For instance in Sudan, found a significant increase in treatment seeking for fever at the community level after introduction of volunteer malaria control assistants [13]. In Burkina Faso, a study that evaluated the impact of adding home management of malaria on health-facility based approach, revealed that the arm that had only health-facility based approach (control group) had low reported cases of fever/malaria (34.1 %) as compared to the intervention group which had reported cases of 87.4 %. In addition, of all malaria cases treated in the intervention group, only 6.7 % were treated at the health facility [14]. This was a clear indication that very few people opted to go to a formal health facility when the child was sick. Similarly, a study in Kenya showed that, only one-third of the under-5 child illnesses had their care sought from the health facilities [15]. In Zambia, there was a significant decrease in the use of health facilities due to mothers’ preference to visit community health workers as their first source of care for most childhood illnesses [16]. It was also shown that long distance to health facilities [17], inadequate income and lack of community education were barriers to prompt treatment of febrile illnesses [18, 19]. These studies suggest that there are important barriers to utilization of the formal health facilities, which offer effective curative and preventive services for many of the diseases of childhood, including malaria.

Poor health care-seeking behaviour for fever among caretakers of under-5 children is still a major concern [9, 11] and this has a huge bearing on the morbidity and mortality in under-5 children. Nevertheless, fever/malaria has remained a major concern of child survival in sub-Saharan Africa despite the existence of effective curative and preventive measures. Although there is availability of health care facilities/qualified personnel in formal health facilities, malaria continues to cause 15–20 % deaths in under-5 children in Zambia [20]. However, these deaths could be prevented if caretakers sought care promptly. This study assessed treatment-seeking behaviour for fever in two rural communities in Zambia.

## Methods

### Study area and participants

The study was conducted in Chivuna and Magoye rural communities of Mazabuka district in Zambia. The target population was children aged 12–59 months. Most of the population in Mazabuka district is rural, consisting of peasants farmers, whose main crops are maize and cotton. Formal education levels are low, rarely going beyond 7^th^ grade. The majority of the settlers in the area are the Tonga speaking people; the largest tribe in the southern part of Zambia. The two areas have flat land with numerous small rivers, but Chivuna is mountainous in few areas. The selection of the two areas was based on their similarities in low treatment coverage during child health week of December 2005, Chivuna (24 %) and Magoye (25 %), catchment population of under-5 children of Chivuna (2404) and Magoye (2431) and numbers of trained health workers at each RHC Chivuna (13) and Magoye (12).

### Study design and data collection

The study was a community based longitudinal study in Chivuna and Magoye. The data for this study was collected during a bigger study which was looking at the “Impact of community-directed treatment on soil transmitted helminth infections in children aged 12 to 59 months in Mazabuka District, Zambia” [21]. The children were randomly selected by systematic sampling, where children were chosen at regular intervals from the sampling frame. Eligible children were those living in the area and aged 12 to 59 months. Trained field assistants using structured questionnaires between July 2006 and November 2007 collected the data prospectively for 1221 children. In which 362 of them had fever and treatment-seeking behaviour was assessed from their caretakers. Hence, the data reported in this study is based on 362 children who had fever. The data was collected from the households of the caretakers on a monthly basis. The caretakers determined the illness (fever) by touching the body. The fever in this study was used as proxy for malaria. This is because at the time this data was collected, the study areas were using fever as a proxy for malaria.

### Data analysis

Data was analysed using STATA (STATACORP, version 12, College Station, Texas, USA). Chi-square test was used for descriptive statistics. A one-sample test of proportion was used to ascertain the statistical significance of two proportions arising from the same sample, with the hypothesis that the proportions were equal. Univariate and multivariate logistic regression (xtlogit model) was used to assess factors associated with early treatment of fever. Confidence interval (CI) of 95 % and a 5 % level of significance was used to assess statistical significance.

## Results

### Basic characteristics of study participants

The total sample reported in this study consisted of 362 children aged between 12 and 59 months with fever, out of the 1221 children recruited. In which, 189 (52 %) children were from Magoye and 173 (48 %) from Chivuna (*p* = 0.4471). The median age of the children was 33 months. There was no statistical difference (*p* = 0.2863) regarding the proportion distribution of males (53 %) and female (47 %) in the study. The median time of delay to seek treatment for fever among the caregivers was 2 days later.

### Assessment of treatment-seeking behaviour (Chi-square test)

The results of univariate analysis to assess the treatment-seeking behaviour for fever are given in Table 1 below, where 41 % of children received medication at home before external treatment was sought. Overall, there was significantly (*p* < 0.0001) higher proportion of under-5 children whose treatment was sought externally compared to those who only received home treatment. Among those that sought treatment from outside the home, a significantly higher proportion of children (64 %) were treated at a health facility as compared to the ones treated by community health workers (18 %) and other sources (friend, relatives, traditional healer or spiritualist) (18 %).

**Table 1:** Characteristics of participants sorted according to health-seeking behaviour

Factors	% (95 % CI)	*P*-value
	Fever (*n* = 362)	
Home treatment with medicine		
Yes	41 (36, 46)	<0.0001^b^
No	59 (54, 64)	
External advice or treatment		
Yes	77 (73, 82)	<0.0001^b^
No	23 (18, 27)	
Source of treatment for fever^a^		
Health facility	64 (58, 70)	<0.0001^aHC/O^
Community Health Worker	18 (13, 23)	
Other sources	18 (14, 23)	

*N* number of children with at least one fever of episode. ^a^Percentages worked on less numbers from the overall due to missing values. ^b^One- sample test of proportions. ^aHC/O^One-sample test of proportions of either Health Facility and Community Health Workers or Health Facility and Other sources

### Health-seeking behaviour for under-five children by study site

**Fig. 1:**
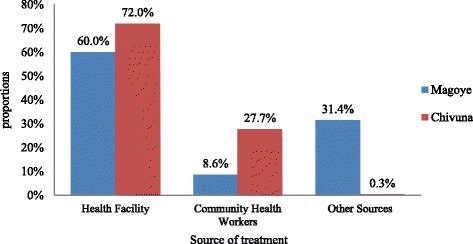
Health seeking behaviour in terms of sources of treatment for fever by study site

In Chivuna, there were more fever episodes (72.0 %) being treated at a health facility in comparison to Magoye (60.0 %). Magoye had a significantly (p<0.001) higher proportion of fever episodes (31.4 %) being treated by other health providers (friends, relatives, traditional healers or spiritualists) as compared Chivuna (0.3 %). The proportion of fever episodes treated by community health workers were significantly (p<0.001) higher in Chivuna (27.7 %) than in Magoye (8.6 %), as shown in Fig. 1.

### Duration between onset of fever and seeking treatment for the fever

Duration of early (≤24 h) and late (> 24 h) seeking of treatment for fever is shown in Table 2. The proportion of caretakers who sought treatment for fever episodes after 24 h of onset were significantly (*p* < 0.0001) higher (58 %) than those who sought treatment within 24 h (42 %). Of those who sought treatment in the first 24 h, 32 % were in the dry season and 68 % in the rainy season. Furthermore, more episodes of fever (56 %) in Magoye had their treatment sought within 24 h compared to those from Chivuna (44 %). Children aged 12–24 months had the highest proportion (34 %) of fever episodes for which treatment was sought within 24 h of onset of fever as compared to those aged 49–60 months (19 %). The proportion of children for whom treatment was sought within 24 h after onset of fever was higher among those who sought treatment from the health centres (67 %) compared to those who sought from the community health workers (17 %) and other sources (16 %).

**Table 2:** Duration between onset of fever and seeking treatment for the fever

Parameters	Timing for treatment		*P*-value
	≤ 24 h	> 24 h	
Fever cases	42 %	58 %	<0.0001^a^
Age (months)			
12–24	34 %	32 %	0.247^b^
25–36	26 %	24 %	
37–48	21 %	25 %	
49–60	19 %	19 %	
No. fever episodes	1083	1307	
Sex			
Male	51 %	50 %	0.657^b^
Female	49 %	50 %	
No. fever episodes	1238	1466	
Sources of treatment for fever			
Health Facility	67 %	65 %	0.290^b^
Community Health Worker	17 %	19 %	
Other sources	16 %	16 %	
No. fever episodes	1304	1814	
Season			
Rainy	32 %	41 %	<0.001^b^
Dry	68 %	59 %	
No. fever episodes	1307	1814	
Location			
Magoye	56 %	47 %	<0.001^b^
Chivuna	44 %	53 %	
No. fever episodes	1307	1814	

^a^One-sample test of proportions. ^b^Chi-square test

### Predictors of early treatment of fever

Univariate analysis (Table 3) showed that, children aged 37–48 months had 17 % (OR 0.83; 95 % CI 0.64, 1.08) reduced chance of having an early treatment for fever as compared to those aged 12–24 months, although the findings were statistically not significant (*p* = 0.176). Female children had a reduced chance of 3 % (OR 0.97; 95 % CI 0.80, 1.16) for early treatment of fever than their male counterparts, although there was no statistical significance (*p* = 0.715). Community health workers and other sources (friends, relative, traditional healers or spiritualist) were 12 % (OR 0.88; 95 % CI 0.70, 1.10; *p* = 0.256) and 8 % (OR 0.92; 95 % CI 0.72, 1.19; *p* = 0.536) respectively less likely to provide early treatment for fever as compared to health facility staff, although the findings had no statistical significance. In dry season, caretakers had 55 % (OR 1.55; 95 % CI 1.31, 1.83) increased chance of seeking early treatment for fever as compared to rainy season, the findings were statistically significant (*p* < 0.001). Children in Chivuna had a significantly (*p* < 0.001) reduced chance of 34 % (OR 0.66; 95 % CI 0.55, 0.79) of having an early treatment for fever as compared to those of Magoye.

**Table 3:** Predictors of early treatment of fever (Univariate and Multivariate analysis)

Variables	Univariate OR (95 % CI)	*P*-value	Adjusted OR (95 % CI)	*P*-value
Age (months)				
12–24	1.00	1.00	1.00	1.00
25–36	1.06 (0.82, 1.37)	0.644	1.01 (0.78, 1.31)	0.935
37–48	0.83 (0.64, 1.08)	0.176	0.79 (0.61, 1.03)	0.085
49–60	1.02 (0.78, 1.35)	0.869	1.01 (0.76, 1.33)	0.966
Sex				
Male	1.00	1.00	1.00	1.00
Female	0.97 (0.80, 1.16)	0.715	0.92 (0.76, 1.12)	0.401
Sources of treatment				
Health facility	1.00	1.00	1.00	1.00
Comm. Health workers	0.88 (0.70, 1.10)	0.256	1.10 (0.93, 1.19)	0.390
Other sources	0.92 (0.72, 1.19)	0.536	0.76 (0.57, 1.01)	0.056
Season				
Rainy	1.00	1.00	1.00	1.00
Dry	1.55 (1.31, 1.83)	< 0.001	1.55 (1.29, 1.85)	< 0.001
Location				
Magoye	1.00	1.00	1.00	1.00
Chivuna	0.66 (0.55, 0.79)	< 0.001	0.60 (0.48, 0.74)	< 0.001

*Abbreviation*: *OR* Odds ratio, *CI* Confidence interval

Multivariate (adjusted) analysis (Table 3) showed that, other sources (friends, relative, traditional healers or spiritualist) had insignificantly (*p* = 0.056) reduced chance of 24 % (OR 0.76; 95 % CI 0.57, 1.01) for early treatment of fever as compared to health facility. Season (*p* < 0.001) and location (*p* < 0.001) were still significantly associated with early treatment of fever. Hence, the best predictors’ model (Table 4), showed that sources of treatment (health facility vs. other sources), season and location were the factors significantly associated with early treatment of fever.

**Table 4:** Adjusted predictors of early treatment of fever (relative risk) from the best model that fits the data well

Predictors of early treatment of fever	OR (95 % CI)	*P*-value
Sources of treatment		
Health facility	1.00	1.00
Community health workers	0.91 (0.73, 1.14)	0.421
Other sources	0.73 (0.56, 0.95)	0.022
Season		
Rainy	1.00	1.00
Dry	1.53 (1.30, 1.80)	< 0.001
Location		
Magoye	1.00	1.00
Chivuna	0.62 (0.50, 0.76)	< 0.001

*Abbreviations*: *OR* Odds ratio, *CI* Confidence interval

## Discussion

Treatment-seeking behaviour for under-five children with perceived fever among rural caretakers in Zambia is still a huge challenge as it was revealed by this study. The delay in seeking treatment can result to disease progression, hence poor prognosis that can ultimately result in death. The study assessed the health-seeking behaviour for under-5 children among caretakers, in which predictors of early treatment were identified.

Our data could have been subject to recall bias as the caretakers were made to remember whether the child had fever in the preceding month. The data did not provide the socio-demographic characteristics of the caretakers, which would have been very cardinal in fully understanding the treatment care-seeking behaviour in our study areas. Hence, this study did not provide the full spectrum of factors influencing health-seeking behaviour. The data for the study was collected in 2007, which may suggest that the situation could have changed. Although the study did not attempt to prove whether there had been fever or not by use of a thermometer, earlier studies [22–24] have shown that caretakers generally have a good understanding of febrile illnesses in terms of types and symptoms. Despite these limitations, fever in children still prompts many caretakers to seek-treatment.

The study revealed that in the rainy season very few fever episodes were treated within 24 h of recognition of symptoms as compared to dry season. The rainy season (November-April) is the time when agricultural activities are at a peak. Hence, the high opportunity costs of time in taking a child for treatment instead of going to the field is likely to lead to delay in seeking early treatment in the rainy season. This compares well with the study by Sauerborn et al. [25] in which it was found that more illness episodes were treated in the dry season than in the rainy season. A study conducted in the same area as this current study by Halwindi et al. [18] revealed that because of filed up streams and hardly passable roads in the rainy season, a number of families were unable to access the health centres. This could be the reason also in this study which led to delay in seeking early treatment in the rainy season. This study has also shown that season was a significant predictor of early treatment for fever among caretakers in our project area. The study further revealed that gender was not a significant predictor of early treatment in our study population. This compares well with studies conducted in Pakistan [26] and Bangladesh [27], where it was found that there was no evidence of gender differences in the care-seeking patterns by the caretakers. It was also shown that children aged 12–24 months were more likely to be treated early as compared to those aged 37–48 months. However, across all age groups, early treatment was not significantly different; possibly suggesting that caretakers apply similar interventions across all age groups. This is in line with a study that was conducted in Northeastern Nigeria [28], where they found that health-seeking behaviour for fever across all age groups was not different.

Most caretakers sought treatment of fever outside the home and the majority of the cases were managed at a health facility. This is similar to the findings of other studies [29–31]. However, this finding is contrary to the report by Muller et al. [32] where most of the fever cases were being managed at home. This difference could have been due to easily accessible health facilities in our study areas and the knowledge caretakers had with regard to formal health facilities as opposed to that of Muller’s. Choosing a health facility is based on the availability and condition of the health facilities, cost, local beliefs and satisfaction with services [30]. However, in the rural areas, a good proportion of caretakers still seek treatment of fever for their children from community health workers (18 %) and other sources (friend, relative, traditional healers or spiritualists) (18 %) as shown by the findings of this study. The cases that went without external treatment existed in a visible proportion and this compares well with an earlier study [33] which found that nearly one-third of the sick children did not have external treatment sought on them. This study also revealed that other sources were significantly associated with delayed treatment of fever in comparison to a health facility and a study in Tanzania [24] showed that self-treatment of febrile resulted in delay to seek medical care. This is a major health concern, as these sources of treatment are likely not to be trained in fever management or treatment. As such, it may result in mismanagement of fever and this, coupled with treatment delay, could heavily contribute to the high rate of under-5 morbidity and mortality more especially in rural communities.

Magoye had more episodes of fever being treated within 24 h of recognition than Chivuna. Magoye is along major high way as such it is possible that accessibility to preventative measures is easier than in Chivuna, which is more inland. This could explain the differences in the two areas. The study also revealed that location was a significant predictor of early treatment of fever episodes. The probable explanation for this is the differences in the health-seeking behaviours in the two study areas. More than half of the caretakers in our study areas did not take any action within 24 h when a child was recognized to have fever, meaning less than half of the children had their treatment sought according to Abuja target of within 24 h of recognition of symptoms [34]. This study compares well with other studies [11, 35], which found a significantly higher proportion of caretakers seeking treatment for fever after 24 h of recognition. This kind of delay in seeking treatment for fever may increase its underlying effects such as morbidity and mortality in under-5 children. Lack of access to treatment/health facilities, poor road networks, lack of drugs in the health facilities, local beliefs and poor socio-economic status of the consumers [19, 36] are some of the reasons for delay to seek treatment care among mothers or caretakers of under-5 children. However, the findings in this study were different from a study in Malawi [8], in which more than half of the children (54.42 %) were treated the same day fever started. The contrariety could have been due to differences in the location and knowledge of caretakers with regard to fever as a dominant feature for malaria. In our study, the median time of seeking treatment was 2 days after recognizing the symptoms, and this was in line with the study conducted in Tanzania [35].

## Conclusion

The study was able to show the health-seeking behavior of caretakers of under-5 children with malaria related fever in Magoye and Chivuna rural parts of Zambia. There was poor treatment seeking behaviour among the caretakers. This could be a contributing factor to morbidity and mortality in under-five children. Source of treatment, season and location was revealed as predictors of early treatment of fever among caretakers and therefore policies aimed at combating poor care-seeking behaviour should not omit to address these aspects. There is also need to enhance interventions aimed at improving the health-seeking behaviour among caretakers such as health education more especially in rural areas.

### Recommendations

The following are the recommendations from this study; there is need for programmes aimed at teaching caretakers of under-5 children on the need of seeking appropriate treatment for fever episodes. Health promotion programmes aimed at specifically increasing the proportion of mothers or caretakers seeking treatment care for the under-5 children from a health facility to above 85 %. The data was conducted in 2006/2007; a lot might have changed, hence, the need to conduct a more recent study.
